# Prevalence of weakness and factors mediating discrepancy between reported and observed leg weakness in people with sciatica

**DOI:** 10.1007/s00586-024-08330-6

**Published:** 2024-06-24

**Authors:** Lucy Dove, Georgios Baskozos, Thomas Kelly, Elaine Buchanan, Annina B. Schmid

**Affiliations:** 1 Nuffield Department of Clinical Neurosciences, John Radcliffe Hospital, The University of Oxford, West Wing Level 6, Oxford, OX3 9DU UK; 2 Oxford Spinal Surgery Unit, Nuffield Orthopaedic Centre, Oxford University Hospitals NHS Foundation Trust, Oxford, UK; 3 Integrated Pain and Spinal Service (IPASS), Berkshire Healthcare NHS Foundation Trust, Berkshire, UK

**Keywords:** Sciatica, Radicular leg pain, Radiculopathy, Weakness, Prevalence

## Abstract

**Purpose:**

To establish the prevalence and agreement between reported and observed leg weakness in people with sciatica. To establish which factors mediate any identified difference between reported and observed leg weakness in people with sciatica.

**Methods:**

68 people with a clinical diagnosis of sciatica, records from spinal service, secondary care NHS Hospital, England, UK reviewed. Primary outcome measures were the sciatica bothersome index for reported leg weakness and the Medical Research Council scale for observed weakness. Agreement was established with Cohen’s Kappa and intraclass correlation coefficient. Potential factors that may mediate a difference between reported and observed weakness included leg pain, sciatica bothersome index sensory subscale, age, hospital anxiety and depression subscale for anxiety.

**Results:**

85% of patients reported weakness but only 34% had observed weakness. Cohen’s Kappa (0.066, 95% CI − 0.53, 0.186; *p* = 0.317)] and ICC 0.213 (95% CI − 0.26, 0.428, *p* = 0.040) both showed poor agreement between reported and observed weakness. The difference between reported and observed measures of weakness was mediated by the severity of leg pain (b = 0.281, *p* = 0.024) and age (b = 0.253, *p* = 0.042).

**Conclusion:**

There is a high prevalence of reported leg weakness in people with sciatica, which is not reflected in observed clinical measures of weakness. Differences between reported and observed weakness may be driven by the severity of leg pain and age. Further work needs to establish whether other objective measures can detect patient reported weakness.

**Supplementary Information:**

The online version contains supplementary material available at 10.1007/s00586-024-08330-6.

## Introduction

Sciatica describes spine-related leg pain of neural origin, and its prevalence could be as high as 43% [1].

At least one third of people with sciatica develop persistent pain lasting one year or longer [2]. The burden of sciatica is considerable, with many people describing total suspension of normal life due to the severity and nature of symptoms [3]. Systematic reviews have failed to show good effect sizes for sciatica treatments, including physiotherapy, pharmacological, injection and surgical management [4–7].

Radicular leg pain is a cardinal feature of sciatica, but a substantial proportion of patients also report symptoms suggesting loss of function such as hypoaesthesia (numbness) and weakness. Leg weakness is a commonly reported symptom in people with sciatica [8–12]. Trials of people with clinically diagnosed sciatica suggest that between 30 and 70% of participants have detectable weakness [13, 14]. Symptoms of leg weakness can persist for two years in up to 25% of people with sciatica [11]. Leg weakness in sciatica is associated with lower physical function and higher emotional distress [8].

Despite the high prevalence and impact of weakness in people with sciatica, little is known about the relationship between the symptom of reported weakness and the clinical sign of observed weakness. Empirically, clinicians often observe that patients report weakness while myotomal muscle testing remains normal. However, to our knowledge there are currently no data on potential discrepancies between reported and observed weakness. This study therefore aims to examine the prevalence and potential discrepancies between reported and observed leg weakness in people with sciatica. In addition, we investigate which factors mediate potential discrepancies.

## Methods

This study is a retrospective cross-sectional study of health records from people with sciatica attending a specialist spinal service of a secondary care NHS hospital in the United Kingdom. Records were extracted by a single author (LD) during a 3.5-month period in 2019; 20% of the records were checked for reliable data extraction by a second author (TK), with no discrepancies in data extraction identified. This study was exempt from ethical review because it was a registered audit (number 7032) with the local NHS Hospital Trust. All patient data were fully anonymised.

Health records of patients over the age of 18 were selected based on the scoring tool of an existing diagnostic model for sciatica [15]. This included unilateral leg pain below the knee; leg pain greater than or equal to back pain; report of any pins and needles or numbness in the involved limb; neurological deficit (motor, sensory or reflex) in any dermatome or myotome of the painful limb, or positive neurodynamic test (e.g., straight leg raise, or slump). We only selected records with a score of 5 out of a possible score of 10, indicating at least 83% predicted probability of sciatica [15]. People with bilateral leg pain were excluded. There were no upper limitations on age, no limit to duration of symptoms or previous history of spinal surgery. Any records indicating the presence of cauda equina syndrome, metastatic disease or other serious spinal pathology were excluded. Any records indicating symptoms of upper motor neuron pathology were excluded.

### Extracted data

Reported weakness was extracted from the sciatica bothersome index (SBI) subscale for weakness. This is a 7-point numeric score ranging from 0 to 6 with anchors: 0 = ‘Not Bothersome’ 3 = ‘Somewhat Bothersome’ and 6 = ‘Extremely Bothersome’. Observed weakness of the affected leg was extracted from clinical notes, using the Medical Research Council (MRC) scale for myotomal strength grading [16]. This scale ranges from 0 (no contraction) to 5 (normal power) and was available in records for lumbar levels L2-S1. If myotomal strength was documented as ‘normal’, observed weakness was given a score of 5. If multiple scores were attributed to different myotomes, the lowest MRC score was used for analysis. A scale of 4+ was scored at 4.5 and 4− at 3.5.

The severity of leg and back pain were separately extracted from 11-point numerical rating scales ranging from 0 (no pain) to 10 (worst pain imaginable). Although there is little evidence in sciatica, the numerical pain rating scale has good validity and reliability for low back pain [17]. Anxiety and depression were both extracted from the respective subscales of the hospital anxiety and depression scale (HADS), found to have good validity in identifying anxiety and depression disorders and assessing symptom severity [18]. Reported numbness or tingling was extracted from the SBI sensory subscale, scored from 0 to 6 as per the weakness subscale previously described.

We further extracted data to describe the patient population. These included demographic measures such as age, gender, body mass index (BMI) and patient reported outcomes such as overall SBI, Oswestry Disability Index (ODI) and the EuroQol EQ-5D index.

### Data analysis

Statistical analyses were computed using IBM SPSS version 28. Agreement between reported and observed measures of weakness was assessed in two ways.

First, reported and observed weakness scores were dichotomised. Reported weakness was dichotomised with any score ≥ 3 considered as reported weakness and any score of ≥ 2 considered as no reported weakness. We considered the cut-off at ≥ 3 (anchor ‘somewhat bothersome’) to be a conservative estimate of reported weakness with potential underestimation of its presence. Observed weakness recorded on the MRC scale was also dichotomised with any score below 5 considered observed weakness whereas scores of 5 were considered as no observed weakness. Agreement between dichotomised reported and observed weakness was tested using weighted Cohen’s Kappa. The strength of agreement was interpreted using conventional divisions [19].

Second, individual continuous data for both reported and observed weakness measures were demeaned, divided by the sample standard deviation and the MRC data-points were multiplied by − 1 to be brought into the same scale as the SBI. Intraclass correlation coefficient two-way mixed effects, consistency, single measurement (ICC (3, 1)) was used to determine inter-rater reliability between normalised reported and observed weakness. ICC values were interpreted using conventional measures [20].

To determine the effects of certain clinical characteristics on the differences of reported versus observed weakness ratings, multiple linear regression analysis was carried out. Weakness data were scaled and normalised before model fit. The dependent variable was the difference between the standardised measures of reported and observed weakness. Four independent variables were selected a priori as potential mediators of the difference between reported and observed weakness: 1. Leg pain severity 2. Age 3. HADS-Anxiety and 4. Reported numbness/tingling recorded on the SBI sensory subscale. Overall significance of the regression model was assessed using ANOVA and significance of estimated coefficients was assessed using t-tests. Estimates are presented alongside their 95% confidence intervals (CI).

Sample size was determined a priori using a simulation over pilot data. We empirically calculated the power of the four fixed effects (leg pain, reported numbness/tingling, age, HADS-Anxiety) in a multiple linear regression setting (y ~ 1 + leg pain, + reported numbness/tingling + Age + HADS-Anxiety), using a simulation where the means and standard deviations of the respective variables were estimated from the data itself. The effect size was set to moderate (0.5), and the residual variance was set to 2^2. After 200 replications, we estimated that a power of 0.8 for all 4 variables, at an α < 0.05 and degrees of freedom equal to sample size—5, can be achieved with a sample size of 67 people.

## Results

We included 68 eligible patient records over a period of 3.5-months. Demographic and clinical characteristics are presented in Table 1.

**Table 1:** Demographic and clinical characteristics (n = 68)

	Mean*	SD*
Age (years)	52.81	15.21
Gender (number female, %)	37	54%
BMI (kg/m^2^)	28.76	5.02
Leg pain (NRS 0–10)	8.26	1.47
Back pain (NRS 0–10)	6.51	2.56
Overall SBI (0–24) median, IQR	19.50	6.00
Reported numbness/tingling (SBI 0–6) median, IQR	5.00	1.75
ODI (0–100)	50.85	18.65
HADS-anxiety (0–21)	9.30	5.37
HADS-depression (0–21)	9.03	5.00
EQ5D index	0.23	0.28
Reported weakness (SBI 0–6) median, IQR	4.00	1.75
Observed weakness (MRC 0–5) median, IQR	5.00	1.00

SD, standard deviation; BMI, body mass index; NRS, numeric rating scale; IQR, interquartile range; SBI, sciatica bothersomeness index; ODI, Oswestry disability index; HADS, hospital anxiety and depression scale; EQ5D,EuroQol 5 dimensions; VAS, visual analogue scale; MRC, Medical Research Council

*Data are expressed as mean (SD) unless labelled otherwise

### Distribution of weakness

**Fig. 1:**
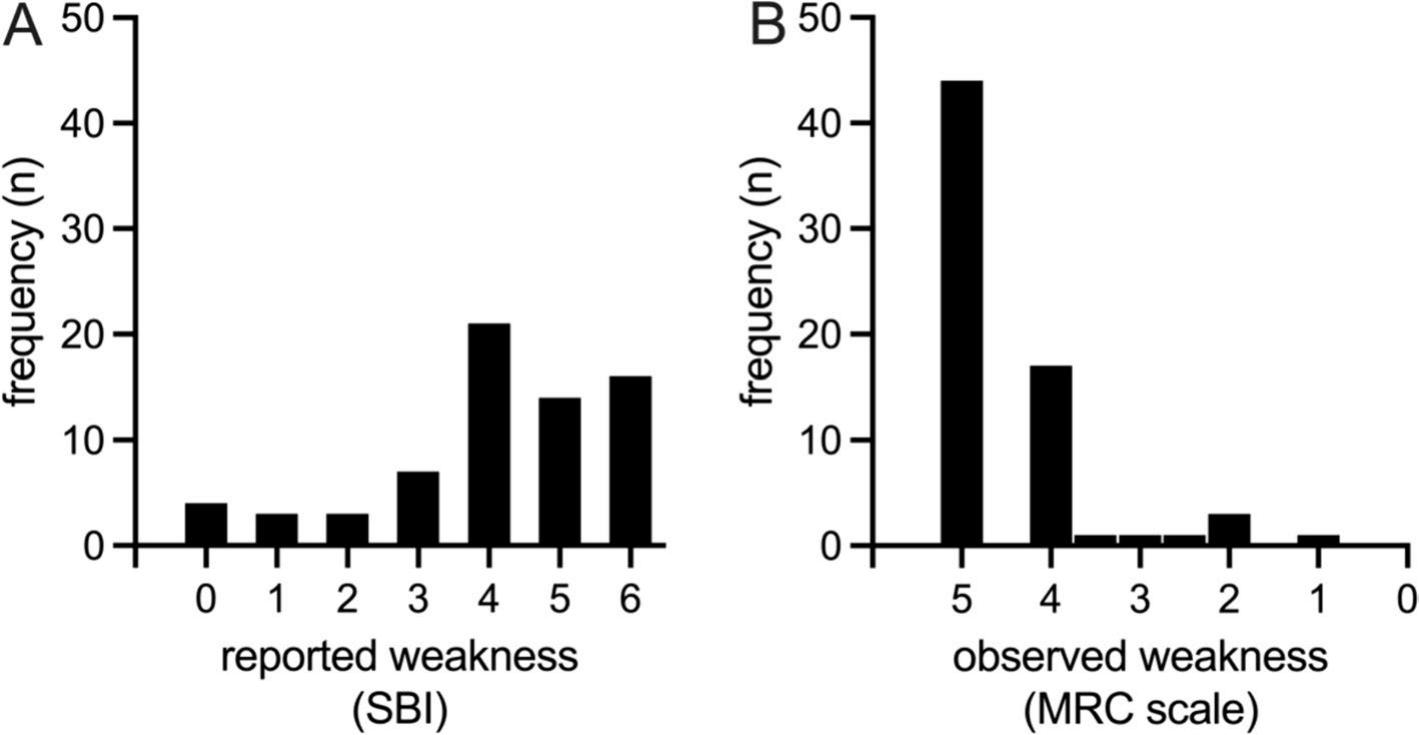
Discrepancy of reported (**A**) and observed weakness frequency (**B**). Reported weakness is graded on the sciatica bothersomeness index (SBI) with higher values representing more weakness. Observed weakness is graded on the Medical Research Council (MRC) scale with lower values representing more weakness

The frequency of reported weakness is illustrated in Fig. 1a. Overall, 58 people (85%) had reported weakness (SBI ≥ 3). Sixteen (24%) of those rated their weakness as ‘extremely bothersome’ (SBI = 6). The frequency of observed weakness is illustrated in Fig. 1b. Overall, 23 people (34%) had observed weakness in lower limb myotomes (MRC < 5).

### Agreement between reported and observed weakness

Agreement between reported and observed dichotomised weakness ratings was only present in n = 21 (31%) of patients (Table 2). Weighted Cohen’s Kappa comparing reported and observed weakness was 0.066 (95% CI − 0.53, 0.186, *p* = 0.317) indicating poor agreement which was not significantly different from zero. Similarly, the ICC (3, 1) comparing scaled reported and observed weakness was 0.213 (95% CI − 0.26, 0.428, *p* = 0.040), indicating poor agreement. The distributions of the standardised measures of weakness are shown in supplemental Fig. 1.

**Table 2:** Contingency table: reported and observed weakness

	Reported weakness (SBI)	No reported weakness (SBI)	Total
Observed weakness (MRC)	21	2	23
No observed weakness (MRC)	37	8	45
Total	58	10	68

SBI, sciatica and bothersomeness index; MRC, Medical Research Council

### Factors mediating discrepancy between reported and observed weakness

The fitted multiple linear regression was significantly better than an intercept only model (R^2^ = 0.203 one-way ANOVA: F (4, 62) = 5.3, *p* = 0.006). The independent variables that predicted the difference between reported and observed weakness were the severity of leg pain (b = 0.281, *p* = 0.024) and age (b = 0.253, *p* = 0.042), Table 3.

**Table 3:** Linear regression predicting discrepancy between reported and observed weakness

	Unstandardised coefficients		Standardised coefficients	*p*-value	95% CI	
	Beta	SE	Beta		Lower bound	Upper bound
(Constant)	− 3.897	1.011		< 0.001	− 5.917	− 1.877
NRS leg pain	0.248	0.108	0.281	0.024	0.033	0.464
SBI sensory	0.088	0.091	0.114	0.335	− 0.093	0.270
HADS-anxiety	0.039	0.030	0.165	0.201	− 0.021	0.099
Age	0.021	0.010	0.253	0.042	0.001	0.041

SE, standard error; CI, confidence interval; NRS, numeric rating scale; SBI, sciatica bothersomeness index; HADS−Anxiety, hospital anxiety and depression scale anxiety subscale

## Discussion

In patients (n = 68) presenting with sciatica to a secondary care spinal service, reported weakness was highly prevalent (85%). Strikingly, reported weakness was 2.4 times more common than observed weakness. Both Cohen’s Kappa and ICC measures of agreement indicated a lack of agreement between reported and observed weakness. Regression analysis revealed that the discrepancy between reported and observed weakness could be predicted by the severity of leg pain and age. The direction of the relationship indicates that with increasing severity of leg pain and advancing age there was an increase in the difference between reported and observed weakness.

In addition to the high prevalence of reported weakness, the stark discrepancy between reported and observed weakness is intriguing. One possible explanation is that the way we measure weakness with the MRC scale is not sensitive enough to pick up nuances of weakness. The MRC scale was originally conceived in 1942, designed to detect substantial changes in weakness such as after traumatic nerve injury [16]. The MRC scale is more reliable and accurate than an analogue scale in the assessment of pronounced muscle weakness at lower MRC grades (0–3) but performs less well when assessing more subtle weakness (MRC grades 4–5) [21]. In our study, and in line with other sciatica cohorts, [2] subtle observed weakness is more common than pronounced weakness. When compared with dynamometry, the MRC scale shows good specificity but lacks diagnostic accuracy and sensitivity in measuring subtle strength deficits [22].

Another possibility is that whilst the MRC scale may adequately measure focal myotomal weakness, it lacks the ability to measure muscle endurance or fatigue which patients may report as weakness. Detection of these less obvious forms of weakness may require enhanced examination such as repeated functional movements [23] or testing in a fatigued state. Further, clinicians generally carry out their neurological assessment with their patient sitting or supine, which may not reflect the situation when reported weakness occurs.

Another possible explanation for the lack of agreement is that reported and observed weakness are not measuring the same parameter, so the symptom is different to the clinical sign. People with sciatica may be striving to describe the overwhelming nature of their symptoms and resultant suspension of activity [3]. Levels of pain-related fear remain high in those people who do not recover two years following onset of sciatica, and conversely, those people who recover from sciatica have low levels of pain-related fear [24]. The loss of confidence in physical activity or attempt to avoid severe neuropathic pain could be placed under the umbrella term ‘weakness’ given the lack of alternative routes to express these symptoms.

Leg pain severity was the strongest predictor of the difference between reported and observed weakness, this relationship has several possible explanations. There is a well-established link between pain and motor function [25]. Pain causes a variety of neuromuscular adaptations that variably affect muscle activity, thereby altering normal motor control. The presence of neuropathic pain could be a key feature, as it has specific qualities distinguishing it from nociceptive pain [26]. Neuropathic pain and its unusual characteristics may well be interpreted as weakness, possibly because the severity and nature of this pain is distinct to previous pain experiences [3]. Further work to profile people with neuropathic pain who describe symptoms of weakness using tools such as the neuropathic pain symptom inventory (NPSI) [27] might advance our understanding of the relationship between neuropathic pain and weakness.

Older age was also a predictor of the difference between reported and observed weakness. Advancing age may simply increase the symptom of weakness due to the established link between sarcopenia and ageing [28]. However, this would be expected to lead to systemic rather than unilateral leg weakness and future studies would need to characterise the location of reported weakness.

We had hypothesised that reported numbness would predict the difference between reported and observed weakness as it represents the sensory correlate for loss of nerve function. However, the SBI sensory subscale was not predictive. An important consideration is that the SBI sensory subscale does not differentiate between numbness and tingling, the latter representing gain of function. Using a screening tool such as pain DETECT [29] that separates the symptoms of numbness and tingling in future work might further dissect any potential relationship between altered sensation and reported weakness.

We included anxiety as a potential mediator of the difference between reported and observed weakness since anxiety is common in those with neuropathic pain [30]. Furthermore, fear avoidant behaviour due to pain is thought to be a factor in the persistence of musculoskeletal pain [31]. Yet again, anxiety was not found to be predictive of the relationship between reported and observed weakness. Although there is a relationship between negative affective emotions and chronic pain, we cannot extrapolate this to anxiety and reported weakness in our cohort. Exploring the relationship between fear avoidance and leg weakness in sciatica may be more useful than examining anxiety per se.

Limitations of this study are the retrospective data collection, which prevented standardisation of the clinical neurological assessment of weakness. In mitigation, all clinicians were in the same spinal triage team, trained in advanced spinal practice. We were also limited by the measures routinely collected in clinics and available on patient records.

### Clinical implications and future directions

Currently, there is a collective failure to provide effective treatment for people with sciatica. Despite the high prevalence of reported weakness, our clinical tests clearly do not observe signs of weakness to the same degree. This leaves our patients with a symptom that their health professional cannot detect, and likely therefore does not recognise, let alone treat. Future work should focus on understanding the exact nature of reported weakness and its meaning for people with sciatica. More work is needed to adequately measure fatigue, lack of endurance or functional movements in this population. A more complete understanding of the signs and symptoms of weakness in people with sciatica could lead to new and improved treatments for this currently underserved group of people.

## Conclusion

This study demonstrates a strikingly high prevalence (85%) of reported weakness in people with sciatica. Notably, there was no agreement in the reported and observed measures of weakness. The variables that predicted the difference between reported and observed weakness were increasing severity of leg pain and advancing age. Future work must accurately describe and measure leg weakness in people with sciatica to fully profile this heterogeneous group. We must accurately understand the variable presentation of sciatica to develop new and effective treatments based on individual symptoms.

## Supplementary Information

Below is the link to the electronic supplementary material.

Supplementary file1 (DOCX 361 KB)
