# Supplementary figures and images for: Prevalence of weakness and factors mediating discrepancy between reported and observed leg weakness in people with sciatica
Source: Eur Spine J. 2024 Jun 24;33(11):4229–34. doi: 10.1007/s00586-024-08330-6 (PMC7616658; doi:10.1007/s00586-024-08330-6)

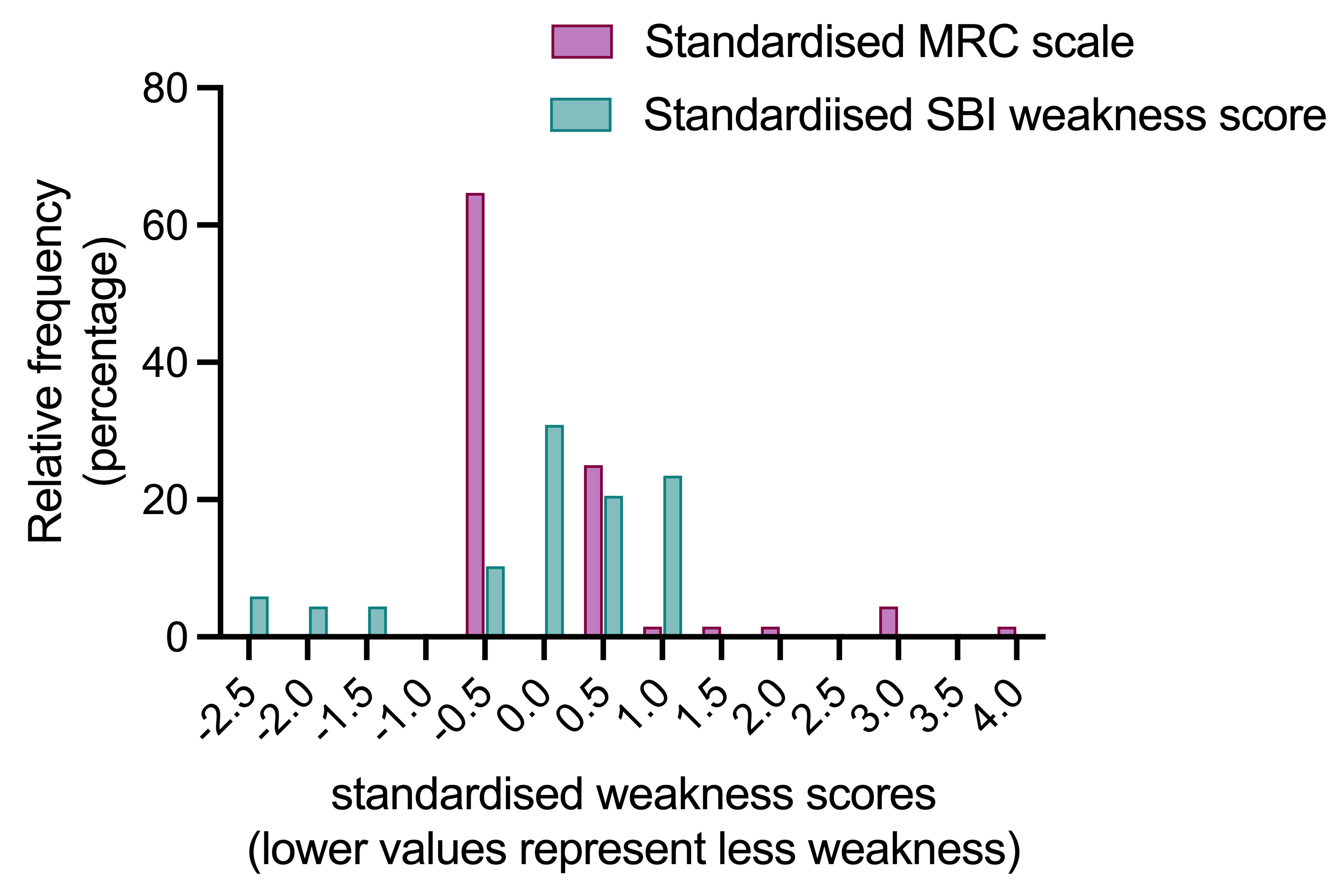


Supplemental Figure 1. Standardised reported and observed weakness.

Supplement: Supplementary file 1 — Supplementary file1 (DOCX 361 KB) [file 586_2024_8330_MOESM1_ESM.docx]
